# Use of LOINC for interoperability between organisations poses a risk to safety

**DOI:** 10.1016/S2589-7500(20)30247-8

**Published:** 2020-10-19

**Authors:** Jacob McKnight, Michael L Wilson, Pamela Banning, Chris Paton, Felix Bahati, Mike English, Ken Fleming

**Affiliations:** Nuffield Department of Medicine, University of Oxford, Oxford OX1 3SY, UK; African Strategies for Advancing Pathology, Denver, CO, USA; 3M Health Information Systems, Salt Lake City, UT, USA; LOINC, Regenstrief Institute, Indianapolis, IN, USA; Nuffield Department of Medicine, University of Oxford, Oxford OX1 3SY, UK; KEMRI-Wellcome, Nairobi, Kenya; Nuffield Department of Medicine, University of Oxford, Oxford OX1 3SY, UK

## Authors’ reply

We thank Alexis Carter and colleagues for the opportunity to revisit this important topic. The use of diagnostic coding systems is a contentious area and represents a further reason why the WHO Essential Diagnostics List (EDL) team should engage the community of specialists in this field before publication of the next edition.

In our original Comment,^1^ we referenced Logical Observation Identifiers Names and Codes (LOINC) as a tool for assisting interoperability, for facilitating patient choice, allowing better national planning, and encouraging better clinical practice. We also mentioned other coding systems such as SNOMED CT and ICD-10 and remain largely impartial as to which systems are used. We did not offer a comparison of these systems, but we encourage the EDL team to review all available options. All coding systems have weaknesses and, importantly, so does the default non-coded system of using highly ambiguous and interchangeable names for tests.

In response to Carter and colleagues, we must address several points. We agree that LOINC is complex because laboratory medicine is complex, and people without appropriate training should not be put in a position to choose the correct code. Including LOINC codes natively in the EDL would help avoid this issue. Although we acknowledge that the LOINC table is a flat structure, LOINC publishes a multiaxial hierarchy (MAH) based primarily on the component (analyte). This is not a strict ontology, but the MAH organises terms based on appropriate categories within a domain. LOINC is updated twice a year, as is SNOMED CT, and these updates are necessary to keep up with the pace of change in laboratory medicine. The survey cited by Carter and colleagues^2^ that shows inaccurate assignment of codes had a 4·7% response rate (90 responses from 1916 laboratories), and although we agree that the findings suggest more investigation might be required, we question how representative this sample is given the strength of their conclusions.

Although it is highly possible that the EDL will benefit high-income countries, it is in the health systems of low-income and middle-income countries (LMICs) that it is expected to have the greatest effect, which is why the open, international, and community-driven approach of LOINC is uniquely suited to the EDL aims. Similarly, we decided to use LOINC in our project in Kenya that inspired our original piece^1^ because it helped us resolve ambiguity in test names that were commonly mistaken by the many untrained staff and patients who used them. The codes helped with clarity and comparison, but they were not used in isolation and we do not advocate for this. Perhaps Carter and colleagues had higher-income settings in mind when they argued for interoperability based on reliance on manual mapping by those with expertise in laboratory medicine. Such individuals are rare indeed in health systems in LMICs.
